# Novel aromatic ring-hydroxylating dioxygenase genes from coastal marine sediments of Patagonia

**DOI:** 10.1186/1471-2180-8-50

**Published:** 2008-03-25

**Authors:** Mariana Lozada, Juan P Riva Mercadal, Leandro D Guerrero, Walter D Di Marzio, Marcela A Ferrero, Hebe M Dionisi

**Affiliations:** 1 Centro Nacional Patagónico (CENPAT-CONICET), Boulevard Brown 2825, Puerto Madryn (9120), Chubut, Argentina; 2 Planta Piloto de Procesos Industriales Microbiológicos (PROIMI – CONICET), Av. Belgrano y Pasaje Caseros, San Miguel de Tucumán (4000), Tucumán, Argentina; 3 Universidad Nacional de la Patagonia San Juan Bosco, Belgrano y 9 de Julio, Trelew (9100), Chubut, Argentina; 4 Programa de Investigación en Ecotoxicología, Intersección Rutas 5 y 7, Universidad Nacional de Luján-CONICET, Luján (6700), Buenos Aires, Argentina

## Abstract

**Background:**

Polycyclic aromatic hydrocarbons (PAHs), widespread pollutants in the marine environment, can produce adverse effects in marine organisms and can be transferred to humans through seafood. Our knowledge of PAH-degrading bacterial populations in the marine environment is still very limited, and mainly originates from studies of cultured bacteria. In this work, genes coding catabolic enzymes from PAH-biodegradation pathways were characterized in coastal sediments of Patagonia with different levels of PAH contamination.

**Results:**

Genes encoding for the catalytic alpha subunit of aromatic ring-hydroxylating dioxygenases (ARHDs) were amplified from intertidal sediment samples using two different primer sets. Products were cloned and screened by restriction fragment length polymorphism analysis. Clones representing each restriction pattern were selected in each library for sequencing. A total of 500 clones were screened in 9 gene libraries, and 193 clones were sequenced. Libraries contained one to five different ARHD gene types, and this number was correlated with the number of PAHs found in the samples above the quantification limit (*r *= 0.834, *p *< 0.05). Overall, eight different ARHD gene types were detected in the sediments. In five of them, their deduced amino acid sequences formed deeply rooted branches with previously described ARHD peptide sequences, exhibiting less than 70% identity to them. They contain consensus sequences of the Rieske type [2Fe-2S] cluster binding site, suggesting that these gene fragments encode for ARHDs. On the other hand, three gene types were closely related to previously described ARHDs: archetypical *nahAc*-like genes, *phnAc*-like genes as identified in *Alcaligenes faecalis *AFK2, and *phnA1*-like genes from marine PAH-degraders from the genus *Cycloclasticus*.

**Conclusion:**

These results show the presence of hitherto unidentified ARHD genes in this sub-Antarctic marine environment exposed to anthropogenic contamination. This information can be used to study the geographical distribution and ecological significance of bacterial populations carrying these genes, and to design molecular assays to monitor the progress and effectiveness of remediation technologies.

## Background

The Southwest Atlantic coast forms the border of one of the most productive marine ecosystems [1]. Extending south of 40°S latitude, the Patagonian coast holds an exceptional biodiversity, sustaining important breeding colonies and feeding grounds for seabirds and marine mammals. One of the most significant threats to the conservation of the overall health of this marine ecosystem is the pollution produced as a result of anthropogenic activities. The main sources of pollution in the region are the release of untreated effluents into coastal waters, fishing and cargo activities as well as oil exploitation and transportation [2]. In particular, anthropogenic hydrocarbons have been detected in sediments at several locations along the Patagonian coast [3,4], and high levels of polycyclic aromatic hydrocarbons (PAHs) were found in marine mammals after an oil spill [5]. The characterization of indigenous hydrocarbon-degrading microbial populations is therefore necessary for a better understanding of natural biodegradation processes in this vulnerable ecosystem and for the successful application of bioremediation technologies.

PAHs are a diverse group of compounds composed of two or more fused aromatic rings, which can have petrogenic, pyrogenic or biogenic origins [6]. The persistence of PAHs in the environment is largely due to their low aqueous solubility, which also results in its association with particulate and sedimentary material and low bioavailability [7]. Typically, the initial step in the aerobic biodegradation of PAHs is the introduction of both atoms of an oxygen molecule at two adjacent carbon atoms of the aromatic nucleus to produce a *cis*-dihydrodiol, a prerequisite for the fission of the aromatic compound [8]. This step is catalyzed by an aromatic ring-hydroxylating dioxygenase (ARHD), a soluble multicomponent enzyme composed of an iron-sulfur flavoprotein reductase, an iron-sulfur ferredoxin and an oxygenase component, whose active site interacts with the aromatic compound [9]. The structure of the naphthalene dioxygenase from *Pseudomonas *putida strain NCIB 9816-4 has been the prototype for all members of the family of ARHDs [10]. The oxygenase component of this enzyme system, naphthalene 1,2-dioxygenase, is composed of two subunits with a α_3_β_3 _structure, each α subunit containing two distinct domains: a Rieske domain that contains a [2Fe-2S] center and a catalytic domain that contains a non-heme ferrous iron ion [10].

Functional marker genes, encoding key enzymes of characteristic metabolic pathways, are often used to specifically target functional guilds of microorganisms because they aid in assigning a likely function for the detected microorganisms in the environment [11]. Although this strategy is able to compensate for some limitations associated with 16S rRNA gene analysis, available information about the actual genetic diversity of functional gene markers is still limited, and presents a strong bias towards sequences that originate from cultured bacteria. In the case of PAH-biodegradation, the generally used gene marker encodes for the large subunit of the catalytic component of the ARHDs, which has been shown to confer substrate specificity [12]. The most extensively characterized group of PAH-dioxygenase genes, a highly conserved group called *nah*-like genes, have been identified in *Pseudomonas *species [13]. More recently, PAH-dioxygenase genes that are evolutionarily different from the *nah*-like genes have been characterized, and the information about these genes is rapidly expanding [8]. Some of these distinct dioxygenase genes were identified in bacteria isolated from the marine environment such as from different strains belonging to the genus *Cycloclasticus *[14,15], *Nocardioides *[16], or *Neptunomonas *and *Pseudoalteromonas *[17,18]. However, the available information concerning PAH-dioxygenase gene diversity in the marine environment is still sparse, in particular from uncultured bacteria. The aim of this study was to expand our knowledge of functional marker genes for PAH biodegradation from the marine environment. We used a culture-independent approach, based on the amplification, cloning and sequencing of ARHD gene fragments from intertidal sediments of Patagonia with different levels of hydrocarbon contamination. We identified eight distinct ARHD gene types, five of them showing low levels of identity with previously identified dioxygenases.

## Results

### Sampling sites and PAH concentrations

**Figure 1:**
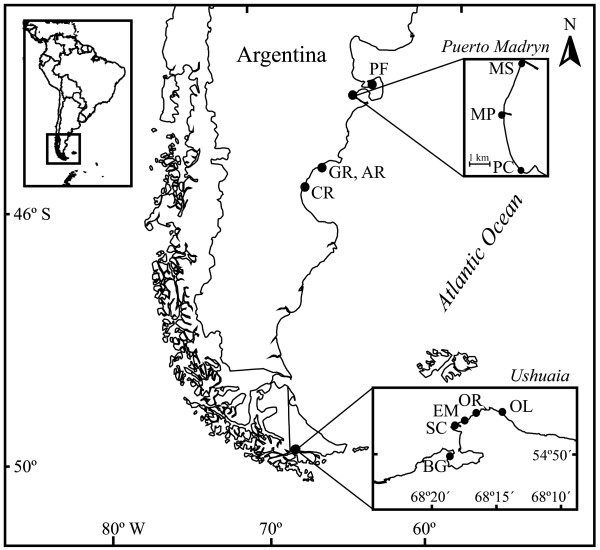
Sampling sites along the Patagonian coast.

Surficial intertidal sediment samples were collected at twelve different locations along the coastline of Patagonia, Argentina. Seven of the sampling sites (north to south: PF, MS, MP, PC, GR, AR and CR) are situated along the eastern coast of Patagonia, at the Chubut Province, next to the Atlantic Ocean (Figure 1 and Additional file 1). In contrast, the five remaining sites (west to east: BG, SC, EM, OR, and OL) are located on the south coast of the Big Island of Tierra del Fuego, next to the Beagle Channel. All sites were sampled once except for OR, which was sampled during three consecutive years. All samples were named by their sampling site and the year in which the samples were retrieved.

PAH concentrations as well as sampling dates are shown in Table 1. Total PAH concentrations of samples MP04, EM06 and OR06 were greater than 1700 μg per kg of dry weight sediment (μg/kg dry wt. sediment), and the number of PAHs detected varied widely between these samples: 1, 2 and 11, respectively. Most of these PAHs exceeded sediment quality guidelines set to protect aquatic life (Table 1, [19]). In addition, four samples (MS05, OR04, OR05 and OL06) showed lower levels of PAH contamination, with total PAH values between 120 and 640 μg/kg dry wt. sediment. All these sampling sites are located in close proximity to piers with heavy shipping traffic or used for loading and unloading of gas oil, fuel oil and gasoline. On the other hand, total PAH concentrations were very low or under the detection limit of the technique in half of the analyzed samples, which included three sampling sites distant from populated areas (PF05, AR06, GR06) as well as four sites situated in close proximity of cities on the Patagonian coast (PC04, CR06, BG04, SC04).

**Table 1:** Concentration of PAHs (μg/kg dry wt. sediment) and sampling dates of intertidal sediments.

	**Sample**														
	**Chubut**								**Tierra del Fuego**						
		
**PAHs**	**PF05**	**MS05**	**MP04**	**PC04**	**GR06**	**AR06**	**CR05**		**BG04**	**SC04**	**EM06**	**OR04**	**OR05**	**OR06**	**OL06**

**Naphthalene**	-	-	-	-	bql	-	-		20	20	-	-	-	35	-
**Acenaphthylene**	-	-	-	-	-	-	-		-	-	-	-	-	42	-
**Acenaphthene**	-	-	-	-	25	-	-		-	bql	-	-	-	44	bql
**Fluorene**	-	-	-	-	-	-	-		bql	bql	-	-	-	88	113
**Phenanthrene**	-	120	2405	-	-	-	24		bql	10	-	640	-	110	49
**Anthracene**	-	-	-	-	-	-	-		-	-	-	-	14	323	-
**Fluoranthene**	-	-	-	-	-	-	-		-	bql	-	-	14	149	-
**Pyrene**	-	-	-	-	-	-	-		-	bql	477	-	-	59	-
**Benzo(*a*)anthracene**	-	-	-	-	-	-	-		-	bql	1326	-	-	-	-
**Indene(1,2,3-*cd*) pyrene**	-	-	-	-	-	-	-		bql	-	-	-	86	402	-
**Dibenzo(*a*, *h*) anthracene**	-	-	-	-	-	-	-		-	-	-	-	-	141	-
**Benzo(*g*, *h*, *i*) perylene**	-	-	-	-	-	-	-		bql	-	-	-	95	333	29
**TOTAL PAHs**	**-**	**120**	**2405**	**-**	**25**	**-**	**24**		**20**	**30**	**1803**	**640**	**209**	**1726**	**191**
**Sampling Date (month/year)**	03/05	04/05	09/04	09/04	02/06	02/06	04/05		08/04	08/04	04/06	10/04	09/05	04/06	04/06

-: not detected; bql: detected at concentrations below the quantification limit (10 μg/kg dry wt. sediment).

The most commonly found PAH was phenanthrene, which was detected in more than half of the samples (Table 1). The highest PAH concentration of a single compound also corresponds to phenanthrene (2,405 μg/kg dry wt. sediment in MP04), followed by benzo(*a*)anthracene with 1,326 μg/kg dry wt. sediment in EM06 sample. In contrast, chrysene, benzo(*b*)fluoranthene, benzo(*k*)fluoranthene and benzo(*a*) pyrene were not detected in the analyzed intertidal sediments.

### ARHD gene libraries

#### Construction of ARHD gene libraries

Clone libraries of PCR-amplified gene fragments coding for the α-subunit of ARHDs were prepared using DNA extracted from coastal sediment samples from Patagonia. High molecular weight DNA (between 10 and 20 Kb) was obtained from all the samples (data not shown). Primers Ac114F and Ac596R [20] produced amplification products of the expected size in half of the analyzed intertidal sediment DNA samples (data not shown). PCR products from seven samples were used to construct the gene libraries Ac-MS05, Ac-GR06, Ac-SC04, Ac-EM06, Ac-OR04, Ac-OR05 and Ac-OR06. These gene libraries were analyzed by restriction fragment length polymorphism (RFLP) using the *Hae*III restriction endonuclease. All clones showing restriction patterns with a low rate of recurrence in the library were sequenced. For RFLP patterns detected at a high frequency, on the other hand, between 7 and 16 clones were randomly chosen per library for sequencing.

To eliminate from the analysis those clones produced as a result of nonspecific priming, the sequences (excluding primer binding sites) were first compared with the GenBank database using the Basic Local Alignment Search Tool (BLAST, [21]). Approximately 75% of the clones carrying inserts of the expected size by gel electrophoresis (362 out of 479 clones) showed significant similarities with α-subunit ARHD gene fragments. Clones carrying amplification products produced due to mispriming events occurred in almost all libraries, and represented up to 50% of the analyzed clones in some samples. The most common nonspecific sequence found in the libraries was 480 bp and showed homology with transcriptional regulatory proteins (data not shown). Nonspecific amplification was not entirely unexpected, because PCR amplifications were performed using a low annealing temperature as previously reported for this primer set (43°C, [20]), in an effort to facilitate binding of the primers even with minor sequence variations at their binding sites.

#### Composition of ARHD gene libraries

**Figure 2:**
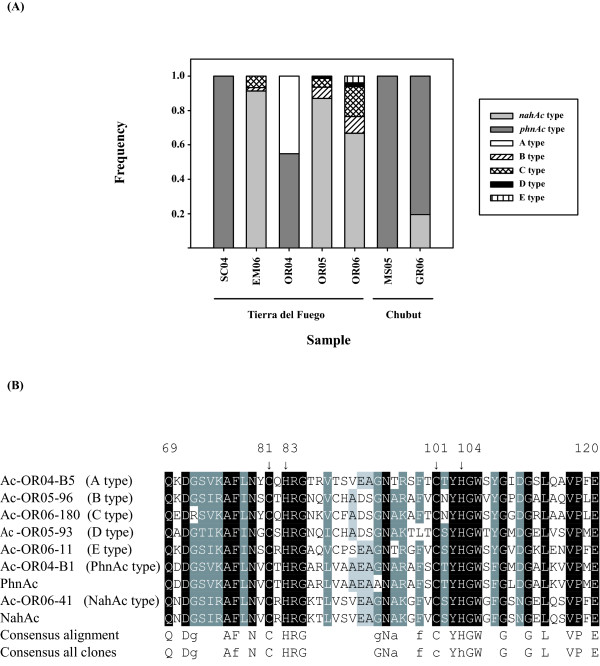
**ARHD gene libraries**. (A) Relative frequency of ARHD gene types recovered in the gene libraries generated with the Ac114F/Ac596R primer set [20]. Gene types were determined by RFLP analysis with the restriction endonuclease *Hae*III, and sequencing. (B) Deduced amino acid sequences of the Rieske-type [2Fe-2S] cluster binding site from sediment clones and reference bacteria. Only one clone for each ARHD type found in the libraries is shown, selected as being the sequence most frequently found in the libraries. PhnAc, *A. faecalis *AFK2 dioxygenase large subunit (accession number BAA76323); NahAc, *P. putida *NCIB 9816-4 naphthalene 1,2-dioxygenase large subunit [AAO64274]. The numbering on the top indicates the deduced amino acid residues of NahAc from *P. putida *NCIB9816-4. Arrows indicate the position of Cys and His residues involved in the coordination of the Rieske [2Fe-2S] center. The last two lines indicate consensus sequences for the shown alignment and for all ARHD sequences found in the libraries, respectively.

Three different insert sizes were found within the 132 sequenced clones that contained ARHD gene fragments: 479 bp, 482 bp and 485 bp. Sequence analysis of all clones clearly separated them into 7 distinct groups, which were defined in this work as different gene types or ARHD alleles. The lowest sequence identity at the amino acid level within a defined group was 94%, and the highest sequence identity between groups was 68.7%. Each library contained between 1 and 5 different gene types (Figure 2A), and this number was correlated with the number of PAHs found in the samples above the quantification limit (*r *= 0.834, *p *< 0.05). One of these gene types, found in four libraries (Ac-GR06, Ac-EM06, Ac-OR05 and Ac-OR06, Figure 2A), showed significant similarities with archetypical *nahAc*-like genes from *Pseudomonas *spp. (97 to 100% DNA and peptide identities with naphthalene dioxygenase from *P. putida *NCIB 9816, [GenBank: AF491307]). All these clones had an insert size of 482 bp. A second gene type, 479 bp long, was found in four different sediment samples (Ac-MS05, Ac-GR06, Ac-SC04 and Ac-OR04) and showed high similarity values with the *phnAc *gene identified in *Alcaligenes faecalis *AFK2 (95 to 98% identity at the nucleotide level and 95 to 99% identity at the amino acid level, [GenBank: AB024945]). Both *phnAc*- and *nahAc*-like genes were detected in the same sediment sample only in one library, Ac-GR06 (Figure 2A).

The five remaining gene types found in coastal sediments, named A to E, were only modestly related (58 to 68% identity in their deduced amino acid sequences) to ARHD sequences from the databases. These sequences had significant matches with ARHD sequences only when using the *tblastx *program of BLAST, which compares the translated query versus the translated database, but not when using the nucleotide-nucleotide BLAST (*blastn *program, [21]). All A, B and E gene types had an insert size of 479 bp, type D gene fragments had 482 bp, and type C inserts were 485 bp long. Alignments of all ARHD nucleotide sequences showed gaps of 3 contiguous bases (data not shown), which were converted into one amino acid gaps in the alignment of the translated sequences [see Additional file 2]. Moreover, two of these gaps were shared by four (types A, B, E and PhnAc-like) or five (types A, D, E, PhnAc- and NahAc-like) groups at the same position.

Figure 2B shows the alignment of the deduced amino acid residues from the Rieske-type [2Fe-2S] cluster binding site of representative clones found in this work, and sequences of two previously identified PAH-dioxygenases (NahAc from *P. putida *NCIB 9816-4 and PhnAc from *A. faecalis *AFK2) closely related to sequences found in the libraries. A number of residues reported to be critical in the Rieske [2Fe-2S] binding site were fully conserved in the alignment shown in Figure 2B (consensus alignment). Nearly all these sites were also fully conserved in the 132 sequenced clones (consensus all clones, Figure 2B).

The number of identified gene types and their relative abundances were used to measure diversity and dominance indices of the ARHD gene libraries (Table 2). The information used to calculate these indices was limited to those sequences able to amplify with this primer set, therefore, it is not possible to make any assumptions about the actual diversity of PAH-degrading bacteria in the communities. The Shannon's diversity index (H) was higher in the Ac-OR06 library, constructed with the sample showing the highest number of PAHs (Table 1). Diversity indices decreased in the following order: Ac-OR04, Ac-OR05, Ac-GR06 and Ac-EM06. Simpson's dominance indices (L) were the lowest in both Ac-OR06 and Ac-OR04 libraries, and increased in the following order: Ac-GR06, Ac-OR05 and Ac-EM06. Despite having only two alleles (*phnAc*-like and novel type A), library Ac-OR04 presented a relatively high diversity value and a low dominance index, since these two gene types were found in almost equal relative proportions. Phenanthrene was the only PAH detected in the OR04 sample (Table 1). In libraries Ac-MS05 and Ac-SC04 only one type of allele was identified, *phnAc*-like, and therefore both showed the lowest diversity index and the highest dominance index (Table 2). Phenanthrene was the only PAH detected in the MS05 sample, while naphthalene and phenanthrene were the only PAHs found at concentrations above quantification limits in sample SC04. Coverage values (C) in the seven ARHD gene libraries were high, ranging from 98% to 100% (Table 2).

**Table 2:** Diversity analysis of ARHD gene libraries from coastal marine sediment samples.

**Library**	***N***	***n***	***H***	***L***	***C (%)***
**Ac-MS05**	34	1	0.00	1.00	100
**Ac-GR06**	72	2	0.71	0.68	100
**Ac-SC04**	16	1	0.00	1.00	100
**Ac-EM06**	92	3	0.50	0.84	100
**Ac-OR04**	20	2	0.99	0.48	100
**Ac-OR05**	77	4	0.73	0.76	98.7
**Ac-OR06**	51	5	1.45	0.48	98.0

*N*, number of ARHD clones screened; *n*, number of detected ARHD gene types; *H*, Shannon's diversity index; *L*, Simpson's dominance index; *C*, library coverage.

#### Phylogenetic analysis

**Figure 3:**
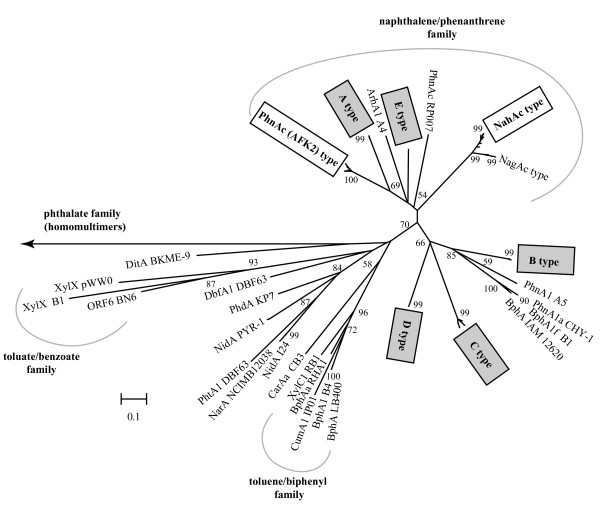
**Phylogenetic analysis of the deduced amino acid sequences of ARHD genes found in the libraries**. The tree was constructed by using the neighbor-joining method in MEGA 3.1 [57] using clone sequences and related proteins, corresponding to positions 45–192 of NahAc in *P. putida *NCIB 9816-4. Bootstrap values were calculated as percentage of 1000 replicates, and values greater than 50% are shown at branching points. The scale bar represents 0.1 inferred amino acid changes per position. Branches containing sequences found in this study are indicated with a rectangle, with shading indicating novel ARHD gene types. The sequence abbreviations (protein name and strain number), species and accession numbers from GenBank are as follows: BphA IAM 12620: *Porphyrobacter sanguineus *IAM 12620 [BAB55875]; PhnA1a CHY-1: *Sphingomonas *sp. CHY-1 [CAG17576]; BphA1f B1: *Sphingomonas yanoikuyae *B1 [ABM91740]; PhnA1 A5: *Cycloclasticus *sp. A5 [BAC81541]; PhnAc AFK2 type: includes PhnAc *A. faecalis *AFK2 [BAA76323], PhnAc *Burkholderia *sp. Eh1-1 [AAQ84686], PhnAc *Burkholderia *sp. Cs1-4 [AAQ84685], PhnAc *Burkholderia *sp. Ch3-5 [AAQ84684] and sequences from this work; ArhA1 A4: *Sphingomonas *sp. A4 [BAD34447]; PhnAc RP007: *Burkholderia *sp. RP007 [AAD09872]; NagAc type: includes NagAc *Ralstonia *sp. U2 [AAD12610], NbzAc *Comamonas *sp. JS765 [AAL76202], NtdAc *Acidovorax *sp. JS42 [AAB40383]; NahAc type: includes NahAc *P. putida *NCIB 9816-4 [AAO64274], NahAc *P. putida *G7 [BAE92156], NahAc *Pseudomonas *sp. ND6 [AAP44288] and sequences from this work; CarAa CB3: *Sphingomonas *sp. CB3 [AAC38616]; XylC1 RB1: *Cycloclasticus oligotrophus *RB1 [AAC44161]; BphAa RHA1: *Rhodococcus *sp. RHA1 [YP707265]; CumA1 IP01: *Pseudomonas fluorescens *IP01 [BAA07074]; BphA1 B4: *Pseudomonas *sp. B4 [CAB93965]; BphA LB400: *Burkholderia *sp. LB400 [AAB63425]; NidA PYR-1: *Mycobacterium vanbaalenii *PYR-1 [AAT51751]; PhtA1 DBF63: *Terrabacter *sp. DBF63 [BAC54156]; NarA NCIMB12038: *Rhodococcus *sp. NCIMB 12038 [AAD28100]; NidA I24: *Rhodococcus *sp. I24 [AAD25395]; PhdA KP7: *Nocardioides *sp. KP7 [BAA94708]; DitA BKME-9: *Pseudomonas abietaniphila *BKME-9 [AAD21063]; DbfA1 DBF63: *Terrabacter *sp. DBF63 [BAB55886]; XylX pWW0: *P. putida *pWW0 [AAA26047]; XylX B1: *Sphingomonas yanoikuyae *B1 [ABM79785]. The arrow corresponds to sequences CarAa CA10: *Pseudomonas resinovorans *CA10 [BAB32766] and Pht3 NMH102-2: *Pseudomonas putida *NMH102-2 [BAA02511].

The amplified ARHD gene fragments were analyzed phylogenetically in order to infer their relationships with other known dioxygenases. Sequences belonging to types A to E formed deeply rooted branches with previously described dioxygenase peptide sequences (Figure 3). The closest relatives of A-type sequences were phenanthrene dioxygenases related to PhnAc from *A. faecalis *AFK2 (67–68% and 78–79% deduced amino acid identities and similarities, respectively). B-type sequences, on the other hand, were most related to BphA from the marine chemoorganotrophic bacterium *Porphyrobacter sanguineus *IAM 12620, formerly *Agrobacterium sanguineum *(68% identity and 82% similarity, [GenBank: BAB55875]). Types C and D were very distantly related (58–60% identity, 75% similarity) to other dioxygenases, and are the most divergent lineages among all these gene types. E-type sequences, on the other hand, were most related to the large subunit of the phenanthrene dioxygenase from *Burkholderia *sp. RP007 (also called PhnAc, 65% identity and 81% similarity, [GenBank: AAD09872]).

From Figure 3, it is not possible to assign potential substrates for these gene types, although types A and E fall, together with all NahAc- and PhnAc-type sequences, inside the naphthalene/phenanthrene family as defined by Gibson and Parales [9]. Sequences from this group, including PhnAc from strains AFK2 and RP007, NahAc from *Pseudomonas *strains and NagAc from *Ralstonia *sp. U2, were classified as Group III ring-hydroxylating oxygenases based on sequence homology [22] and fall into Group III of the Batie classification system, based on the constituent components and the nature of the redox centers of the enzymes forming the electron-transport chain [23,24]. Gene types B, C and D, on the other hand, were excluded in the phylogenetic tree from previously defined families based on substrate (toluate/benzoate, toluene/biphenyl, and phthalate families) [9].

**Figure 4:**
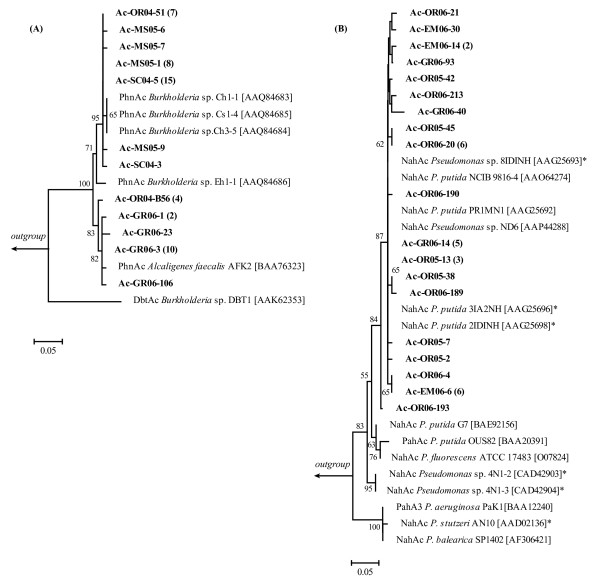
**Phylogenetic analysis of PhnAc- and NahAc-like sequences recovered from the libraries and their closest relatives from the database**. (A) PhnAc-type (*A. faecalis *AFK2) clones and related sequences and (B) NahAc-type clones and related database sequences. Sequences identified in this study are depicted in bold, and named as: the primer set used (Ac) followed by the sample and year from which they originated and their number in the clone library. Clones with = 99% identity in their deduced amino acid sequences were grouped, and the numbers in parentheses refer to the number of clones represented by this sequence. Reference sequences from GenBank include the accession number in brackets. The asterisks show marine isolates. Outgroups used were PhnAc from *Burkholderia *sp. RP007 [AAD09872] for PhnAc-type (*A. faecalis *AFK2) sequences (A) and PhnAc from *A. faecalis *AFK2 [BAA76323] for NahAc-type sequences (B). Bootstrap values were calculated as percentage of 1000 replicates, and only values greater than 50% are shown. The scale bar represents 0.05 inferred amino acid changes per position.

To illustrate the relationships between PhnAc and NahAc type sequences and their close relatives, clone sequences clustered into 99% deduced amino acid identity groups were aligned with reference sequences from the databases, and two phylogenetic trees were created (Figure 4A and 4B). The five PhnAc type (AFK2) ARHD sequences deposited in the databases at the present time were aligned with PhnAc-like sequences found in coastal sediments (Figure 4A). Deduced amino acid sequences of all *phnAc*-like clones found in sediment libraries exhibited 94.5 to 99.3% sequence identity with the PhnAc sequences identified to date from pure cultures. Phylogenetic analysis of PhnAc-like sequences retrieved from sediment libraries and the ones from pure cultures revealed two clades, one containing phenanthrene dioxygenase sequences from the isolates *Burkholderia *sp. strains Cs1-4, Ch1-1, Ch3-5 and Eh1-1 and a second one with PhnAc from *A. faecalis *AFK2 (Figure 4A). Representatives from both clades were found in the Ac-OR04 library. On the other hand, all Ac-SC04 and Ac-MS05 PhnAc-like sequences clustered within the *Burkholderia *clade, while all Ac-GR06 PhnAc-like gene fragments clustered within the *A. faecalis *AFK2 clade (Figure 4A).

NahAc-type sequences formed two distinct, highly supported groups, one of them included sequences from *Pseudomonas stutzeri *strain AN10, *Pseudomonas aeruginosa *PAK1 and *Pseudomonas balearica *SP1402 and the other clade included sequences belonging to *P. putida *strains NCIB 9816-4, G7, OUS82 and other related *Pseudomonas *isolates (Figure 4B). Both groups include sequences from marine isolates (marked with asterisks in the tree) and have been described previously as AN10 and C18 groups respectively [25]. Only sequences from the C18 cluster were detected in coastal sediments. This is not unexpected since the AN10 group was most probably not targeted by the primers used in this work (all sequences show mismatches with at least one of the primers used).

#### Construction and analysis of *phnA1*-like ARHD gene libraries

A primer set was designed to target *phnA1*-like ARHD sequences from bacteria belonging to the genus *Cycloclasticus*. This primer set successfully amplified a ~500 bp fragment from *Cycloclasticus pugetii *PS-1 (ATCC 51542), which was confirmed as a *phnA1 *gene fragment by sequencing. Amplifications with this primer set also resulted in products of the expected size in samples MP04, GR06, AR06, SC04, OR04 and OR05 (data not shown). Samples PF05, MS05, CR05, OR06, EM06 and OL06 were not tested with this primer set. The amplification products of samples SC04 and OR05 were cloned and analyzed by RFLP analysis with the *Rsa*I restriction endonuclease. As all the analyzed clones showed restriction patterns identical to the one produced by the amplification product of *C. pugetii *PS-1, 12 clones from both libraries were randomly chosen for sequencing. Deduced amino acid sequences of these clones had identity values between 98.6 and 100% with sequences from *Cycloclasticus *isolates [GenBank: BAC81541, AAC95148, AAC95146, AAD04819 and ABF56510]. The lowest identity value corresponds to one clone carrying a transcription termination site. This clone could represent a non-functional pseudogene or a PCR artefact.

### Other primer sets analyzed

Primer sets targeting other known PAH-dioxygenase genes were designed and tested with sediment DNA. The groups targeted were *Neptunomonas *and *Pseudoalteromonas *[GenBank: AF053735, AF053736, AF295036], *Burkholderia *sp. RP007 and related genes [GenBank: AF061751, AY540615–AY540620, AY154358, AY154360, AY154362, AY154365 and AY032936–AY032937] as well as *nagAc*-like genes [GenBank: AF036940, AF252550, AF169302, PSU49504, AF379638, BSU62430, DQ167474, AY367788, AY568278, AB066442, AF448048, AF448053, AB066443–AB066445 and AY194931]. We were not able to successfully amplify these gene fragments from the sediment DNA (data not shown). However, it is important to note that these genes could be present in the sediment but below the detection limit of the technique, or the genes present in these samples might be too divergent to be recognized by the primer sets.

## Discussion

In this study, we characterized ARHD genes in intertidal sediments from the Argentinean coast of Patagonia. We used a culture-independent approach, screening catabolic gene libraries prepared with two primer sets, one previously designed using ARHD gene sequences from PAH-degrading bacteria commonly found in terrestrial environments [20], and a second one targeting marine PAH-degrading bacteria belonging to the genus *Cycloclasticus *(this study). Wilson *et al*. [20] designed primers Ac114F and Ac596R using four archetypical *nahAc *type sequences from various strains of *Pseudomonas *isolated from soil, and the *phdAc *gene sequence from *Comamonas testosteroni *GZ39, which was isolated from river sediment [26]. Six degeneracies had to be included in the primers to accommodate this last sequence [20]. Amplicons generated with this primer set include the information for an almost complete Rieske domain and about 40 amino acids into the catalytic domain of the large subunit of the enzyme, based on the structure of naphthalene 1,2-dioxygenase from *P. putida *NCIB9816-4 [10]. To the best of our knowledge, this is the first time these primers have been used to detect ARHD gene fragments from marine environments. Primers Ac114F and Ac596R have been used to amplify PAH-dioxygenase genes using environmental samples from different terrestrial habitats, including groundwater [20,27], seep sediments [28] and soil [29-32].

Seven different ARHD gene types were amplified from intertidal sediment samples using these primers, five of them (types A to E) representing dioxygenase genes with no close relatives in the databases. Their deduced amino acid sequences contain the consensus pattern of bacterial ring-hydroxylating dioxygenase alpha-subunits: C-x-H-R-[GAR]-x_(7,8)_-[GEKVI]-[NERAQ]-x_(4,5)_-C-x-[FY]-H (PROSITE: PS00570, [33], Figure 2B). This consensus includes the two cysteines and two histidines (Cys81, Cys101, His83 and His104 in *P. putida *NCIB9816-4, [10]) that are involved in the coordination of the iron ions in the Rieske [2Fe-2S] center of many dioxygenases [34] as well as Arg 84, important for the formation of hydrogen bonds between subunits [10,35]. Other residues not included in the consensus but also important for the interaction between subunits are also conserved in all these novel gene types. These residues include Tyr103, Trp106 [10,35,36] and Val117 [10].

Gene types A to E were only detected at two sampling sites (OR and EM), located on the coast of Ushuaia city, the southernmost city of the world with a population of approximately 45,000 inhabitants. This region has a humid and temperate-cold climate, average water temperatures of 4.5°C in winter and 9°C in summer, and a tidal range of less than 1 m [37]. Site OR is situated next to a fuel wharf and site EM is located 0.3 km west of OR site, and in close proximity to two piers. GC-MS analysis of the samples retrieved at these sites indicated the presence of a variety of PAHs, in particular in the OR06 sample. Although A-type genes were abundant in the Ac-OR04 library, gene types B to E were detected at very low frequencies. It is then possible that organisms carrying these alleles are not very abundant in these sediment microbial communities. These libraries may even overestimate their actual abundance, as rare target genes can potentially be enriched due to the rehybridization of the most abundant PCR products during the amplification [38,39]. On the other hand, their frequencies could also be underestimated by the primer sequences and the conditions used for the amplification. Further studies are needed to identify the hosts of these alleles, to reveal the ecological significance of these populations in these sub-Antarctic sediments and to discover their actual biogeographic distributions.

In four libraries the most abundant clone type was closely related to *phnAc*-like genes from *A. faecalis *AFK2 [GenBank: AB024945] as well as *Burkholderia *sp. strains Cs1-4, Ch1-1, Ch3-5 and Eh1-1 [GenBank: AY367784–AY367787]. All these strains have the ability to degrade phenanthrene [40,41]. They were isolated from soil and, to the best of our knowledge, this is the first report of the presence of *phnAc*-like genes (*A. faecalis *AFK2) in marine environments. Archetypical *nahAc*-type genes were also rather abundant in the sediment libraries, accounting for more than half of the analyzed ARHD clones. Their sequences are closely related to *nahAc *genes from *Pseudomonas *strains isolated from heavily polluted marine sediments of Barcelona, Spain (Figure 4B, [25]). This allele has also been found in *P. putida *strains isolated from Antarctic marine sediments ([GenBank: AJ496392–AJ496395] Bosch, R., Lalucat, J. and Rossello-Mora, R., unpublished), indicating that cold adapted PAH-degrading organisms can host this gene type. Moreover, evidence of horizontal gene transfer of the *nahAc *gene has been reported in *Marinobacter hydrocarbonoclasticus*, an extremely halotolerant organism isolated from marine sediments [42]. Organisms related to *Marinobacter *were enriched in long term microcosms prepared from Arctic sea ice with crude oil [43], suggesting that psychrophilic representatives of this genus are important in hydrocarbon degradation in cold marine environments. It is then possible that hydrocarbon-degrading bacteria other than *Pseudomonas *may carry the *nahAc*-type genes detected in coastal sediments of Patagonia.

We also evaluated the presence of *pnhA1*-like genes from the *Cycloclasticus *group in coastal sediments. Only one full sequence [GenBank: AB102786], and three partial sequences [GenBank: AF093000, AF092998 and AF053737] were available in the database as of January of 2004. As these sequences were practically identical, we designed a primer set specific for this allele using the *phnA1 *full-length sequence from *Cycloclasticus *sp. A5. Only one sequence has been added to the database since, the *phnA1 *gene from *Cycloclasticus *sp. P1 [GenBank: DQ501245], with a 98.8% similarity at the nucleotide level with the sequence from the A5 strain. This sequence shows differences in critical positions with the newly developed primer set, and would probably not be amplified in the conditions used in this study. Using this primer set, we detected *phnA1*-like genes in most of the analyzed intertidal sediments from Patagonia. Furthermore, DGGE analyses of naphthalene and phenanthrene enrichments using the OR05 sample showed the presence of 16S rRNA genes with high similarity to *Cycloclasticus spirillensus *(98–100%, M. Ferrero, unpublished results). These results suggest that PAH-degrading *Cycloclasticus *are present in coastal sediments of Patagonia. Marine organisms belonging to the genus *Cycloclasticus *appear to be ubiquitous even in non-contaminated sediments [44-46]. It has been suggested that these bacteria play an important role in the degradation of petroleum PAHs in the marine environment [47,48]. They become abundant in oiled seawater microcosms incubated at 4°C, suggesting an important role for these bacteria in PAH-biodegradation even at low temperatures [46]. Therefore, it is not surprising to find these PAH-degrading bacteria in the coastal sediments of Patagonia. To determine if populations carrying *phnA1*-like genes are the major players during PAH biodegradation in marine sediments, where these compounds tend to accumulate due to their low water solubility, it is essential to assess their relative abundance and their level of activity when compared with other indigenous PAH-degrading populations.

## Conclusion

This work expands the current knowledge concerning the diversity of ARHD genes in the marine environment. Despite using two primer sets designed from a limited range of targeted PAH-dioxygenase genes, we detected representatives of eight lines of descent of dioxygenase genes in coastal sediments of Patagonia. The identification of PAH-degrading microorganisms and the development of molecular tools to rapidly identify changes in their populations are fundamental to study the ecological mechanisms governing the intrinsic bioremediation of these harmful aromatic compounds in coastal environments.

## Methods

### Sediment samples

Surficial sediments (0–3 cm) were sampled using acrylic cores with an inner diameter of 4.4 cm. Sampling was performed along the low tide line at seven to ten random points in each sampling location, and the composite samples were placed in sterile glass flasks and stored at 4°C during transport to the laboratory. Each sediment sample was mixed thoroughly and stored at -80°C for the preparation of clone libraries, or at -20°C for chemical analysis.

### Chemical analyses

The PAH concentrations were determined using gas chromatography – mass spectrometry techniques according to US EPA 8100 [49] and Dean [50]. Briefly, sediments were mixed with anhydrous Na_2_SO_4 _in dichloromethane and extracted by sonication for 12 h. Extracts were filtered across a 0.45 μm fiberglass filter and concentrated with a rotary evaporator to a final volume of 1 ml. Two μl were injected into a GC injection port operating in splitless mode. A Shimadzu gas chromatograph 17A V 1.3 model with mass spectrometer QP 5050A and an MS Workstation Class 5000 (Shimadzu Corp., 1999) was used. Samples were analyzed using SIM mode for optimal sensitivity scanning only the quantification ions for each PAH. Quantification was performed by the external standard method (Restek, Bellafonte, PA). Spiked sediment showed recovery of 100 ± 7%.

### Extraction of DNA from coastal marine sediments

High purity, high molecular mass DNA was purified in duplicate from 0.5 to 0.8 g wet weight sediment using the FastDNA^®^SPIN kit for soil (Q-BIOgene, Carlsbad, CA), according to the manufacturer's instructions with the following modifications: samples were homogenized three times for 50 s at approximately 5,000 rpm (speed at high setting) with 1 min intervals using a mini-beadbeater Biospec (Bartlesville, OK) and sediment DNA was eluted in 150 μl 10 mM Tris-HCl pH 8.0 prepared in molecular biology grade distilled water (Invitrogen, Carlsbad, CA). The two extractions per sample were combined before further analysis.

### Amplification of ARHD genes from sediment DNA

Alpha-subunit ARHD gene fragments were amplified using two primer sets: Ac114F/Ac596R [20] and Cyc372F (5' CGATGAGTTGGATAGAGATTCG 3')/Cyc854R (5' GGTTCTCCAAGGTTCTCTG 3') (this study) that target *phnA1*-like genes identified in *Cycloclasticus *spp. PCR amplifications were carried out in 25 μl-reactions containing 50 mM KCl, 10 mM Tris-HCl pH 9.0, 0.1% (v/v) Triton X-100, 1.5 mM MgCl_2_, 0.2 μM dNTPs, 0.5 μM of each primer and 1 U of T-PLUS DNA polymerase (Inbio-Highway, Tandil, Argentina) for all libraries, except for Ac-MS05 (see below). Template concentration was optimized for each sample, and the DNA concentration showing the most intense amplification product of the expected size by gel electrophoresis was used to construct the library (1 to 2 μl of extracted DNA per 25 μl reactions). PCR reactions were performed on a PTC-100^® ^thermal cycler (MJ Research, Waltham, MA). The programs used for the amplification were as follows: 5 min at 94°C, 40 cycles of 30 s at 94°C, 30 s at 43°C (Ac114F/Ac596R) or 50°C (Cyc372F/Cyc854R), and 30 s at 72°C, and a final elongation step of 15 min at 72°C. In the sample MS05, the previously described mix produced multiple amplification products ranging from 1.5 kb to 100 bp with the Ac114F/Ac596R primer set (data not shown). As the amplification with the AccuPrime™ SuperMix II (Invitrogen, Carlsbad, CA) produced only one product of the expected size, this mix was used to construct the library Ac-MS05. The PCR program was the same as above except for the extension step, which was performed at 68°C according to the manufacturer's instructions. All PCR runs included a negative control reaction with no added DNA, and a positive control with the PAH-dioxygenase gene from *Pseudomonas putida *ATCC 17484 (Ac114F/Ac596R) or *C. pugetii *PS-1 ATCC 51542 (Cyc372F/Cyc854R). PCR products were separated by electrophoresis in 1.5% (w/v) agarose gels with 0.5× TBE stained with 0.5 μg/ml ethidium bromide [51].

Other tested primer sets targeted PAH-dioxygenases from *Neptunomonas *and *Pseudoalteromonas *(NP45F 5' GGAGGTTTATGGTGGCTTAC 3'/NP560R 5' GATAAATTCTGGCACGATCAGC 3'), *Burkholderia *sp. RP007 (RP007-296F 5'GCTTCGCCTGCAATTATCATG 3'/RP007-770R 5'ACGTCATATAGCGCACCGATC 3') and *nagAc*-like genes (nagAc108F 5' CTGGCTTTTTYTSACYCATG 3'/nagAc858R 5' CCGRACATCRCCGATTTC 3').

### TA cloning, screening and sequencing of ARHD gene libraries

For the construction of libraries Ac-SC04, Ac-OR04, Ac-MS05, Cyc-SC04 and Cyc-OR05, products from a single PCR reaction were immediately cloned into the pCR^®^4.0 vector (TA Cloning kit for sequencing, Invitrogen, Carlsbad, CA) without further purification, according to manufacturer's instructions. In libraries Ac-OR05, Ac-OR06, Ac-EM06 and Ac-GR06 four PCR amplifications were combined, and the band of the expected size was excised from 1.5% agarose gels, purified using a GENECLEAN^® ^III kit (Q-BIOgene, Carlsbad, CA) and cloned. Library clones were screened by RFLP analysis. Amplified clone inserts were digested with 5 U of the restriction endonuclease *Hae*III (Promega, Madison, WI) or *Rsa*I (Promega), followed by electrophoresis in 2% (w/v) NuSieve 3:1 agarose gels (FMC BioProducts, Rockland, ME) with 0.5× TBE and 0.5 μg/ml ethidium bromide [51]. Clones representative of each RFLP pattern were sequenced. The inserts were sequenced commercially at Macrogen (Seoul, Korea) from primer sites located on the vector.

### Analysis of ARHD gene libraries diversity

Diversity and similarity calculations were based only on ARHD sequences. The sequence information obtained from each RFLP pattern generated from ARHD gene fragments was used to define gene types or alleles. Indices calculated included: library coverage (*C*) [52], or the portion of a clone library of infinite size that was sampled, calculated as *C *= 1 - *yx*^-1^, where *y *is the number of ARHD gene types that occurred only once, and *x *is the number of clones screened; the Shannon diversity index [53], calculated by use of the equation *H *= -Σ (*ni/N*)(log_2_*ni/N*), where *ni/N *is the proportion of clones belonging to each ARHD type relative to the total number of clones; the Simpson's dominance index [54], calculated by use of the equation *D *= Σ [*ni *(*ni*-1)/*N *(*N*-1)] where *ni *is the number of clones belonging to each ARHD type and N is the total number of clones. Shannon and Simpson's indices were calculated by using PRIMER v5 software [55].

Correlations between variables were analyzed using the bivariate two-tailed Pearson correlation in SPSS version 11.5 (SPSS Inc., Chicago, IL).

### Phylogenetic analysis

To construct the phylogenetic trees, clone sequences were clustered into 99% deduced amino acid identity groups, and only one representative sequence was kept for further analyses. The number of sequences that this clone represents is depicted between parentheses, next to the clone name. Deduced amino acid sequences (162 positions) were aligned with reference ARHD sequences obtained from the NCBI database using ClustalX 1.81 [56]. Phylogenetic trees were constructed using the neighbor-joining algorithm in the Molecular Evolutionary Genetics Analysis software (MEGA 3.1, [57]). To test the inferred phylogeny, a bootstrap test with 1,000 replications was used.

### Nucleotide sequence accession numbers

The sequences determined in this study have been deposited in the EMBL nucleotide database under accession numbers AM930890–AM930971 and AM930511–AM930517.

## Authors' contributions

ML carried out part of the laboratory work, participated in data analysis, and wrote part of the manuscript. JPRM and LDG carried out part of the laboratory work. WDDM performed the chemical analysis of the sediments. MAF assisted in the preparation of the manuscript. HMD designed the study, carried out part of the laboratory work and wrote part of the manuscript. All authors read and approved the final manuscript.

## Supplementary Material

Additional file 1 **Supplementary information of sampling sites**. Latitude, longitude and description of sampling sites. Click here for file

Additional file 2 **Sequence alignment of ARHDs**. Deduced amino acid sequence alignment of the ARHD gene fragments found in the libraries, and related sequences. Click here for file
