# Supplementary material for: Novel aromatic ring-hydroxylating dioxygenase genes from coastal marine sediments of Patagonia
Source: BMC Microbiol. 2008 Mar 25;8:50. doi: 10.1186/1471-2180-8-50 (PMC2364624; doi:10.1186/1471-2180-8-50)
Supplement: Additional file 1 — Supplementary information of sampling sites. Latitude, longitude and description of sampling sites. [file 1471-2180-8-50-S1.doc]

### Additional file 1 – Supplementary information of sampling sites

| **Argentinean Province** | **Sampling Sites** | **Description** | **Location** |
| --- | --- | --- | --- |
| **Chubut** | **PF** | Fracasso Beach, located in the protected natural area of Valdez Peninsula | 42º 25’ 32.23” S  64º 07’ 02.58” W |
|  | **MS** | Storni Pier, main port of Puerto Madryn city. Used for cargo and fishing ships | 42° 44’ 13.81” S  65° 02’ 11.90” W |
|  | **MP** | Piedra Buena Pier, Puerto Madryn city. Used for large cruise ships from December to April and small vessels all year | 42º 45’ 47.81” S  65º 02’ 02.42” W |
|  | **PC** | South of Puerto Madryn city, tourist beach | 42º 47’ 03.15” S  65º 00’ 31.82” W |
|  | **GR** | Gravina Peninsula, unpopulated area | 45° 08’ 45.58” S  66° 27’ 32.11” W |
|  | **AR** | Aristizábal Peninsula, unpopulated area | 45º 11’ 54.90” S  66º 30’ 58.29” W |
|  | **CR** | Comodoro Rivadavia city port. Used for cargo and fishing ships | 45º 51’ 41.16” S  67º 28’ 05.07” W |
| **Tierra del Fuego** | **BG** | Golondrina Bay, west of Ushuaia city. Near the Airport | 54º 50’ 18.24” S  68º 19’ 04.42” W |
|  | **SC** | Beach located next to the Saint Christopher tug, aground in Ushuaia Bay since 1957. Only recently, in 2004, the fuel oil was removed from its tank and fuel leaking from the tug over the years was evident | 54º 48’ 33.37” S  68º 18’ 33.09” W |
|  | **EM** | 0.3 km west of Orion Plant, Ushuaia city. Exposed to heavy shipping activity | 54º 48’ 19.19” S  68º 17’ 39.05” W |
|  | **OR** | Orion Plant fuel wharf, Ushuaia city. Operates mainly with gas oil, fuel oil and gasoline | 54º 48’ 16.71” S  68º 17’ 23.67” W |
|  | **OL** | Sediments on Ushuaia Bay, next to Olivia River. East of Ushuaia city | 54º 47’ 56.54” S  68º 13’ 55.03” W |
